# The value of health service-based research to health service organisations: a qualitative study with senior health service executives

**DOI:** 10.1186/s12961-024-01149-z

**Published:** 2024-05-31

**Authors:** Angela L. Todd, Nicholas Petrunoff, Michael Frommer, Don Nutbeam

**Affiliations:** 1 Sydney Health Partners, Level 2, Charles Perkins Centre, The University of Sydney, Sydney, NSW 2006 Australia; 2 The Sax Institute, Level 3/30C Wentworth St, Glebe, NSW 2037 Australia

**Keywords:** Health care organisations, Research translation, Health services research, Research priorities, Health service leaders

## Abstract

**Background:**

Research evidence has demonstrably improved health care practices and patient outcomes. However, systemic translation of evidence into practice is far from optimal. The reasons are complex, but often because research is not well aligned with health service priorities. The aim of this study was to explore the experiences and perspectives of senior health service executives on two issues: (1) the alignment between local research activity and the needs and priorities of their health services, and (2) the extent to which research is or can be integrated as part of usual health care practice.

**Methods:**

In this qualitative study, semi-structured interviews were conducted with senior health leaders from four large health service organisations that are members of Sydney Health Partners (SHP), one of Australia’s nationally accredited research translation centres committed to accelerating the translation of research findings into evidence-based health care. The interviews were conducted between November 2022 and January 2023, and were either audio-recorded and transcribed verbatim or recorded in the interviewer field notes. A thematic analysis of the interview data was conducted by two researchers, using the framework method to identify common themes.

**Results:**

Seventeen health executives were interviewed, including chief executives, directors of medical services, nursing, allied health, research, and others in executive leadership roles. Responses to issue (1) included themes on re-balancing curiosity- and priority-driven research; providing more support for research activity within health organisations; and helping health professionals and researchers discuss researchable priorities. Responses to issue (2) included identification of elements considered essential for embedding research in health care; and the need to break down silos between research and health care, as well as within health organisations.

**Conclusions:**

Health service leaders value research but want more research that aligns with their needs and priorities. Discussions with researchers about those priorities may need some facilitation. Making research a more integrated part of health care will require strong and broad executive leadership, resources and infrastructure, and investing in capacity- and capability-building across health clinicians, managers and executive staff.

## Background

The successful transfer of discoveries and innovations from research into health care has resulted in improvements in clinical practice and health services organisation over many decades [1]. Research translation has led to better diagnoses, better understanding of health conditions, better options for addressing health problems, and successful implementation of optimal health care in different settings with different patient groups. Effective research translation drives improvements in the quality, safety and effectiveness of health care and health outcomes [2].

Despite these clear benefits, the systemic translation of beneficial innovations from research into improved clinical practice too often fails and/or takes too long to implement at the scale required to achieve widespread benefit [3]. This results in a paradox—while new research-based knowledge has proliferated, health benefits have not occurred at a pace or scale that could reasonably be expected. The overall connection between evidence and health care practice has remained broadly static leading to what Braithwaite and colleagues describe as the 60-30-10 Challenge: 60% of care on average is in line with evidence- or consensus-based guidelines; 30% is some form of waste or of low value, and 10% is harmful. They argue that this 60-30-10 Challenge has persisted for three decades [4].

The reasons for this are multi-dimensional. The translation of research into sustained system-wide change in a complex health system is not a simple, linear process. Numerous barriers have been identified reflecting organisational and financial impediments and inherent clinician conservatism, as well as gaps in the necessary capacity and capability to undertake translational research and successfully implement changes in health policy and practice [5–9].

The interests of curiosity-driven researchers and the priorities of health services are often misaligned. Researchers often seek answers to questions that are not of immediate relevance in health service delivery. Important, researchable questions that have more immediate relevance to health organisations and patients are not proportionately recognised or addressed [10]. Examples include the management of patient flow in hospitals, the best ways to provide care to patients with complex needs, and effective transitions from hospital to community care [11, 12]. Accelerating the translation of research evidence into better health care is more likely when it is connected to the needs and priorities of health services [13].

In response to these challenges, several authors and organisations have identified the need to embed research in health care in more systematic and sustainable ways to match researcher capacity and health system needs and priorities, and to facilitate research translation [14–18]. To this end, large-scale structural reforms have been introduced in different parts of the world. These include the establishment of formal partnerships between research institutions and health care organisations through academic health science centres (AHSCs) in North America and the UK and, more recently, research translation centres (RTCs) in Australia [8]. More focused research funding programs targeting health system priorities have also been introduced, for example, through the Agency for Healthcare Quality and Research (AHQR) in the USA [19], the National Institute for Health and Care Research (NIHR) in the UK [20], and the Medical Research Futures Fund (MRFF) in Australia [21].

At a more local level, interventions to support effective research translation have included greater engagement between researchers and decision makers [22–24], improving access to research findings [25], and skills development for translation among researchers and users of research [26, 27]. Attempts have also been made to clarify the priorities of research users, especially clinicians and health service managers responsible for commissioning services, as well as patients and consumers. However, these attempts have not been as successful as hoped [28]. The choice of research topics tends to reflect the priorities and requirements of agencies that award peer-reviewed research grants, the curiosity and interests of individual researchers, or clinical trial opportunities led by industry. Although some of the national and local strategies identified above have sought to re-orient research to health priorities, the motivations and incentives for researchers to respond to the more immediate priorities of those responsible for health services delivery remain limited. While health service organisations directly invest in research, and have substantial internal capacity for it, the linkage of research capacity to health services priorities remains weak.

Most previous examinations of these issues have been based on assumptions about the receptiveness of health service leaders to innovation, the use of evidence, and the level of their support for health and medical research. In this study we examined these assumptions by exploring the experiences and perspectives of senior health service leaders on two issues: the alignment between local research activity and the needs and priorities of the health services for which they have responsibility; and the extent to which research is or can be embedded in health care to encourage more rapid and widespread use of research evidence by those responsible for delivering health services.

## Methods

### Study design and setting

Semi-structured interviews were conducted with senior health leaders from four large health service organisations that are members of Sydney Health Partners (SHP), one of Australia’s nationally accredited research translation centres [29]. These centres support collaboration between health service organisations, universities, and independent research institutes, and are broadly similar to academic health science centres and networks in North America and the UK. They aim to improve the alignment of research capacity with health service needs and to speed up the translation of research findings into evidence-based health care. At the time of this study, the four health service organisations within SHP together provide health services for approximately 2.7 million adults and children across metropolitan Sydney through 16 hospitals and various community and population health services.

### Participants

We purposively selected a sample of 22 senior health service leaders with a broad range of decision-making roles across the four health service organisations. SHP sent an initial email to all 22 potential participants, informing them of the study and seeking permission for SHP to forward their contact details to the research team. The research team sent a second email to respondents, formally inviting them to participate in an interview and directing them to an online participant information statement and consent form.

### Data collection

An interview guide was developed and pilot-tested with five senior health service staff. No changes were made to the protocol because of the pilot work, so these five interviews were included in the analysis of results. The semi-structured guide used the following statements to stimulate discussion with interviewees on the two issues of alignment of research with health service priorities, and embedding research in health care:

- **Statement 1:** Not enough research is sufficiently aligned with the real challenges facing our health system.
- **Statement 2:** There should be a greater emphasis on research being embedded within health services (as opposed to being produced externally).

The interview guide was sent to the consenting health service leaders prior to their scheduled interviews. Interviews were conducted by experienced qualitative researchers from an independent research organisation between November 2022 and January 2023, and took approximately 30 min. In response to participant preferences, interview data were recorded either in interviewer field notes, or by audio-recording which was transcribed verbatim with the participant’s consent.

### Analytical methods

Two authors (AT and NP) conducted a thematic analysis of the interview data using the framework method [30] and with reference to the consolidated criteria for reporting qualitative studies (COREQ) [31]. This process consisted of five steps:

1. Each coder independently reviewed interviewer notes and transcripts and created research diaries recording overall impressions and ideas for initial topic areas.
2. Three transcripts were randomly selected and independently reviewed by each coder to identify topic areas and themes within them. These were then compared between coders for consistency. This process was repeated with an additional three transcripts to formulate an analytical framework.
3. The analytical framework was applied by indexing further transcripts to assess the appropriateness and consistency of topics and themes. The coders maintained a research diary to record iterations of the analysis, and discussed and adjusted the framework as necessary.
4. A separate framework matrix chart was created for a more detailed analysis of the transcripts and notes pertaining to the two statements. Data were synthesised under the topics and themes, creating summaries of the literal meanings of the data and highlighting illustrative quotes. The matrix charts, tables and quotes were then reviewed by a third researcher (MF).
5. The research team interpreted the data, examined connections across topics and themes, considered the generalisability of the findings to other health systems, and resolved disagreements to reach consensus.

### Ethics approval

Ethics approval for the project was granted by the Sydney Local Health District Human Research Ethics Committee (approval number 2022/ETH01259).

## Results

Of the 22 senior health service leaders invited to participate, 17 (77%) consented to be interviewed. The remainder did not respond to the invitation. The participants comprised chief executives (*n* = 4); directors of clinical governance or medical education (*n* = 3); executive-level directors of nursing (*n* = 2); executive directors of medical services (*n* = 2); directors of research (*n* = 2); an executive-level director of allied health (*n* = 1), and three others in diverse executive leadership roles (e.g., chief information officer). All participants were members of the senior executive team of the four health organisations.

Following analysis of the interview data, two main topic areas emerged under each statement. They were as follows:

- *Under Statement 1*: Balance in health research; Alignment of research with health care priorities.
- *Under Statement 2*: Essential elements of embedding research in health care; Overcoming silos at system and health service levels.

Within each topic area, several themes were identified. Tables 1 and 2 summarise the topic areas and themes for each of the statements, and further detail and illustrative quotes follow.

**Table 1:** Topic areas and themes under Statement 1: Not enough research is sufficiently aligned with the real challenges facing our health system

Topic: Balance in health research
*Theme: curiosity- and priority-driven research* • All research is important, but a re-balancing of curiosity- and priority-driven research is needed (Related: Topic 1.2, Theme 1.2.1) • Some types of research (e.g., health services research) were covered insufficiently
*Theme: supporting research activity in the health system* • Health service staff need support to conduct research, e.g., processes; capacity building; awards; funding opportunities; partnerships with academia (Related: Statement 2 responses) • Challenges to conducting research within the health system include complexity of administrative processes; lack of follow-through after successful completion of projects; lack of access to funding; culture of risk aversion; unclear pathways for intellectual property and commercialisation; inadequate access to health data and IT systems; shortage of research implementation scientists and research translation expertise

Topic: Alignment of research with health care priorities
*Theme: identifying health priorities and developing research questions* • It is essential to communicate priorities and involve researchers, clinicians and community in developing priorities and research questions. Both researchers and health service leaders may need help (e.g., from academics) in articulating their priorities and research questions
*Theme: fit between research and health care* • Several research and health care delivery factors have led to a lack of fit or alignment between research and health care priorities
*Theme: lack of profile of research in health care* • Much research activity in health services is not noticed by those outside research teams, who often also do not understand the research

**Table 2:** Topic areas and themes under Statement 2: There should be a greater emphasis on research being embedded within health services

Topic: Essential elements of embedding research in health care
*Theme: leadership* • Sustaining embedded research requires executive support, a clear strategy, an organisational culture that integrates research into health care, and financial investment
*Theme: systems and processes* • Systems and processes to support research exist but would need to be expanded to facilitate system-wide embedded research, and known barriers would need to be addressed
*Theme: supporting staff, building capability and capacity, providing incentives and opportunities* • Embedding research requires involvement of clinician leaders, investments in building capacity and capability, and providing incentives, rewards, and dedicated time for research
*Theme: supporting research translation* • Embedding research includes actively applying research outcomes so that staff can see why research is worthwhile

Topic: Overcoming silos (at system level and health service level)
*Theme: separation between health services and academia* • Embedding research is challenging because research and health care delivery mostly happen in separate systems
*Theme: fragmentation within and between health services* • Silos within and between health service organisations create difficulties for collaborative, larger scale research activities addressing common health problems
*Theme: whose job is research?* • Research needs to be viewed as a part of the role of all health staff

## Statement 1: Not enough research is sufficiently aligned with the real challenges facing our health system

### Topic 1.1: Balance in health research

#### Theme 1.1.1—Curiosity- and priority-driven research

All health service leaders affirmed that research was very important for the quality of health service delivery. They all respected the contributions of, and continuing need for, investigator-led and curiosity-driven research as well as priority-driven research. However, they felt that the balance should be adjusted towards priority-driven research.

Research driven by health services is very important. In the past, research has tended to be driven by the individual interests and ideas of researchers, but now the big issues in health services development are being driven more by research, and it is important that this can happen. (Director of Research)

Several interviewees noted that knowledge gaps in health service delivery, where research could assist, were more likely to be identified by clinicians and health service managers than by researchers. Specific examples included questions on how services are run (e.g., at the ward level), how to apply implementation science to address specific clinical questions on prevalent care problems (e.g., incontinence and pressure injury), and how to enhance the functional wellbeing of the ageing population. One director noted that research on such questions is less likely to produce prominent advances and major publications. This diminishes researcher interest and access to competitive funding.

#### Theme 1.1.2—Supporting research activity in the health system

Most health service leaders were firmly committed to supporting research infrastructure, capability and capacity in their own organisations, whether or not they saw research as contributing explicitly to decision-making or service delivery. Much of the discussion of the need for further investment focused on generating more support for clinical staff and managers to do research through dedicated research time or availability of research support staff.

I think the reality is, as with all things, there’s just not enough money, and also we need to continue the investment in our managers as well as our clinicians to support them to undertake research. (Chief Executive)

Several challenges to conducting research were also identified by health service leaders, including: complex administrative and approval processes; inadequate access to health data and IT systems; a culture of risk aversion; lack of or limited access to funding; lack of follow-through or ongoing support for ‘successful’ projects; unclear pathways for intellectual property and commercialisation; and a shortage of research implementation scientists and research translation expertise to facilitate research uptake into health care.

### Topic 1.2: Alignment of research with health care priorities

#### Theme 1.2.1—Identifying health priorities and developing research questions

Many of the health service leaders expressed some difficulty in framing their problems in researchable terms. They were often reluctant to approach researchers for help in problem-solving because they did not feel confident in articulating questions with sufficient clarity.

Management and clinicians need to be better at articulating their needs for knowledge and communicating their strategic priorities. Researchers investigate highly specific, complex issues, and their findings are not necessarily applicable to prevailing health service problems. (Other Executive)

Engage researchers in the health services management and policy processes and decisions. Change the thinking of health services executives on what research is (as distinct from other forms of enquiry such as audit) and continually remind them of what research can do. (Director of Medicine)

Two interviewees also mentioned the importance of consumer and patient driven research and the need to include consumer voices in identifying research priorities.

… there's a stronger role that consumers can play… a lot of the digital health research that I see is very much [about] investment, return on investment… we said in the business case that we were going to reduce the length of stay… Where is the consumer? What does the patient's family think? What does that particular culturally and linguistically diverse community think about that solution? That's just as important research. I'd love to see more of that. (Other Executive)

#### Theme 1.2.2—Fit between research and health care

Many interviewees observed a lack of connection between researcher priorities and health service challenges. The urgency of many of the issues that arise in health care often leads to an operational focus in health service management and a tendency to seek solutions reactively. Because of time pressures in health services, the pace of demands often outstrips the relatively slow pace of research (although several interviewees noted that the COVID-19 pandemic created exceptions to this).

While it is recognised that the benefits of research may not be manifest for a long time, it seems reasonable to ask whether researchers have a perspective that their research will lead to clinical outcomes? Do they start talking to ’implementers’ early enough? Should we be looking at implementation earlier? (Director of Clinical Governance or Education)

In addition, researchers and health service leaders tend to be preoccupied with different aspects of health fields. For example, in the field of precision medicine, researchers tend to be interested in biomedical and clinical questions, while health service leaders want to know how to incorporate precision medicine into service delivery at scale. Some interviewees perceived a need for assistance in connecting effectively with researchers, identifying a potential brokering role for organisations with expertise in the transfer of research evidence.

#### Theme 1.2.3—Lack of profile of research in health care

Some interviewees argued that research activity and the contributions of research to health care were often invisible to, unknown to, or not well understood by many health care professionals. To achieve a better alignment of research with priorities, it was suggested that the profile of health research should be elevated. This requires greater involvement of researchers with health service management as well as greater exposure of health managers to research processes so that they can see the potential benefits of research to health care.

## Statement 2: There should be a greater emphasis on research being embedded within health services

### Topic 2.1: Essential elements for embedding research in health care

#### Theme 2.1.1—Leadership

All health service leaders recognised the value of embedding research in health care. Most considered that, taken as a whole, the research enterprises in their organisations were not systematically or culturally embedded in health care. They identified several essential elements to achieve this. The importance of leadership was emphasised most consistently, referring to advocacy for research by Chief Executives and other senior staff as an integral and essential activity within health service organisations. Mechanisms for this included embedding research in organisational culture, listing research in corporate strategies, making explicit budget allocations for research, stating research explicitly in job descriptions, and drawing attention to research in performance appraisals:

… we need absolute agreement at executive levels that research is going to inform service delivery. (Director of Research)

there also has to be a matching culture in the staff, that research is something that you do when you're working in the health system. (Chief Executive)

if research is to be embedded … it has to be funded, and strategies are needed to support it. (Chief Executive)

#### Theme 2.1.2—Systems and processes

Interviewees noted that embedding research within health care depends on the support of a range of enabling systems and processes, some of which may have to be expanded or created anew.

… there needs to be an ecosystem and there needs to be policies, there needs to be investment, there needs to be tool sets, forums and meetings. (Other Executive)

there're certain capabilities and infrastructure you need to be able to do this well …. having the capabilities to be able to characterise our cohorts, organise those cohorts, …. high performing support mechanisms, ethics, governance, startup. (Director of Research)

Some interviewees noted that health service organisations’ capacity to invest in research varied, and those with constrained resources had to limit their aspirations:

…at a local level, we try to create opportunities for innovation… whether that's through quality improvement activities, innovation awards. (Chief Executive)

Several interviewees commented on well-known barriers to research in existing systems and processes, notably barriers to accessing data for research. The increasing bureaucratic burden of compliance with governance and risk management for health research, imposed by both the health system and funding agencies, was also a major impediment.

I think organisations could do a lot better to make conducting research easier for people who work in the organisations. I know plenty of colleagues who go, I’m not interested in doing any research because it’s so bureaucratic and the whole ethics process, the governance processes around it are soul destroying … (Director of Clinical Governance or Education)

#### Theme 2.1.3—Supporting staff, building capability and capacity, and providing incentives and opportunities

Senior health service leaders acknowledged that embedding research in health care depended on significant investments to build capability and capacity across their organisations. Although health service organisations did fund research activity and infrastructure, more was needed to support widespread embedding of research. This included leveraging the efforts of clinical leaders already engaged in research to support colleagues locally, partnering with academics, challenging managerial staff to embrace research in their work roles, and offering practical incentives, rewards, and dedicated time to staff for research.

we've been very fortunate to have …. [two named senior clinicians] …work constantly with the team to encourage more and more surgeons to become involved in research. (Director Research)

it's often really hard to allow a nurse off the ward to go and do some work for two or three hours on something that they're really interested in, and they are interested in research, but we can't allow that. (Director of Nursing)

Some interviewees pointed out that investment in clinician researchers has tended to favour medical research and given less attention to other fields.

I think Australia has a strong tradition of medical curiosity-driven research. The role of nurses has been very small, sort of boxed up…I think that’s sort of changing now that nursing’s started to find its feet a little bit more ……especially in the implementation science space where we are now looking, you know, at what we’re doing with our patients. (Director of Nursing)

#### Theme 2.2.4—Supporting research translation

Several interviewees argued that health service organisations generally had limited internal expertise in research translation. They asserted that efforts to embed research needed to be complemented by activities that showed staff the value of research for health care delivery, particularly research oriented towards health service needs and priorities.

There is a need for us to become much more sophisticated in applying evidence in a more individualised and nuanced way rather than…the one-size-fits-all way. (Director of Research)

at what point do we stop and think about what needs to be taken into the system (research evidence)? How do we take things into the system? - that's still not happening … The system's not set up to do that. (Director of Research)

### Topic 2.3: Overcoming silos at system and health service levels

#### Theme 2.3.1—Separation between health services and academia

Interviewees consistently noted a significant separation between academic research institutions and health service organisations. They emphasised that these two “worlds” need to be brought closer together to support embedding research in health care. Structural and systems-based support and partnerships with academia are especially needed for the new frontiers of health research. Strategies suggested to bridge the divide included creating opportunities for clinician researchers and conjoint appointments.

‘every system is perfectly designed to deliver the results you get’. If you set up a system where health care providers and academic researchers are in completely separate organisations, it's no surprise that it may be difficult to marry the two up because you've set up your system to be separate. (Director of Clinical Governance or Education)

I think…we need to marry up a lot more with our research partners and that's something that I'm… investing myself into by becoming… closer with the deans of the universities who have…that affiliation with us. (Director of Nursing)

#### Theme 2.3.2—Fragmentation within health services

Many interviewees also observed that health service organisations and units within them were siloed. These divisions create difficulties for collaborative, larger scale research activities that might identify and address, for example, health problems common to different clinical units within the one hospital or shared by neighbouring health services.

we're all working on our distinct projects that are our distinct strategic goals and it's because we're not all the one service, even though we're under the one umbrella. You don't know what your colleagues are doing unless you're in those meetings. (Director of Clinical Governance or Education)

So, I'm aware of the same project in three different districts all asking the same research question, and a lot of duplication … (Other Executive)

One interviewee highlighted the need to move away from the singular researcher model:

often we view a research [idea]… as a very…individually centric idea or [as having] to be delivered by [a specific] team. And as we're starting to work through the new… frontier of precision medicine, I keep asking the question, ‘Why? Why is this for one person to implement if we actually want to liberate this to other disease groups? And what does that model look like for us? What do advanced therapeutics teams look like, and what is the imperative of the health system to be ready to implement?’ …we are not able to do this at scale. (Director of Research)

#### Theme 2.3.3—Whose job is research?

Although many interviewees had common views on how to support the embedding of research in health care, views on who might be responsible and/or involved in the process were mixed. Some interviewees believed that research should be a shared role across many health service leaders, while others saw research as someone else’s responsibility.

I think that the challenge is also getting managers across the whole system to realise that research is something that is relevant to them and not just to clinicians. (Chief Executive)

I'll be honest, the intention of my team is never really going to be research in its focus because we're very much sort of boots in mud in, you know, problem solving ... (Director of Clinical Governance or Education)

Although there were expected differences in orientation and emphasis between the interviewees, overall the responses reflected a high level of support for research of all types from basic discovery research through to applied research with more direct application to health services. Where variation in response was observable, it often reflected personal experience and engagement with researchers, and the different history, infrastructure and expertise that existed between and within the health organisations.

## Discussion

The health service leaders in this study were affiliated with Sydney Health Partners (SHP), a research translation centre that now comprises five large health service organisations that service a combined population of 3.1 million people, a university with one of the largest health and medical faculties in the Southern Hemisphere, and several independent medical research institutes [29]. Together, the Partners form a uniquely large-scale health and medical research ecosystem with extraordinary research capability. The health service leaders from this Partnership shared a clear commitment to health research. Their feedback reflects the fact that there is a high volume of research activity across the Partnership, but it is not evenly distributed across health organisations. Although there are some exemplary research relationships within clinical practice, research is not systematically embedded as part of ‘usual business’ in everyday health care across the organisations. Feedback from health leaders also suggested most of the research activity was investigator-led and curiosity-driven rather than overtly aligned with the needs and priorities of the health services.

Advocacy for greater integration of research in health care focuses on the benefits of making health care challenges more visible to researchers, building trusted working relationships between researchers and potential users of research, and facilitating better translation and use of innovations arising from research in health care delivery [14–18]. The responses from the health service leaders demonstrate widespread support for these aims, but also show that many considerations and ‘critical elements’ are needed to achieve widespread and sustainable integration of research in health care. These critical elements include not only the executive leadership evident from the responses received, but also an enabling culture that permeates all levels of health service organisations including frontline clinical staff. Sustained integration of research into service delivery requires resourcing and infrastructure, as well as professional development and workforce capacity building, and a range of operational supports and processes including streamlined access to data and research governance approval. Many of these same elements have been identified by others working to embed research into health care systems for decades [32]. Although these key factors exist in each of the SHP organisations, they vary significantly between organisations reflecting historical patterns of investment and differences in priorities.

The health service leaders who participated in this study held the highest executive positions in large complex health service organisations yet acknowledged that they needed help to communicate effectively with research partners and build collaborations relevant to health service priorities. They expressed difficulties finding time to invest in such relationships, and highlighted a general disconnection between researchers’ usual incentives, priorities and ways of working, and health services’ strategies, imperatives and operational pressures. While they agreed these changes needed to be led ‘from the top’, several of the health leaders recognised that the wider executive team and middle managers in the organisation were more likely to be active agents in this process. One of the key functions of establishing research translation centres like Sydney Health Partners (and similar academic health science centres in other countries, for example King’s Health Partners in the UK [33]) is to create environments and opportunities that support closer collaborations between researchers, health professionals and increasingly patients and consumers, to support more research that matters to the potential users of research.

In addition to actions that can be implemented by health service organisations, research funding agencies have opportunities to influence researcher behaviour. For example, grant programs are being directed towards research addressing health service priorities and programs that help build health service research capacity and capability. In Australia, the Medical Research Futures Fund (MRFF) was established to do this. Its stated objectives include funding research that addresses national health priorities; building stronger relationships between researchers, health care professionals, governments and the community; and facilitating implementation of health innovations arising from research discoveries [21]. To date, the distribution of the MRFF has not always followed these objectives. A review is currently in progress to improve the strategic directions and coordination of health and medical research funding in Australia. The outcomes of the review could help to attune the MRFF more closely to its mission [34].

Our study involved the most senior health leaders from four large public health organisations and achieved a high participation rate (77%). Framework analysis was used to interrogate the data rigorously and systematically. This method of qualitative data analysis was developed specifically for policy relevant research such as ours [35]. However, this small qualitative study has limitations. All health service leaders were from metropolitan services that had strong and longstanding partnerships with local universities and contained major teaching hospitals. It is therefore likely that those participating in the interviews had greater awareness of, and engagement with academic colleagues than would have been the case in other health organisations. It is also possible, because of these factors, that the views expressed by health service leaders may have reflected the expectations of individuals in senior leadership roles. However, the consistency of key themes across different roles and organisations suggests otherwise. We adhered to a well-established qualitative design and semi-structured interview focused on two key questions, but for practical reasons some of the interviews were audio recorded and others relied on interviewers’ notes. The subsequent steps towards interpretation of the data were the same for both interview modalities.

## Conclusions

Universal endorsement of the value of research among health service leaders was tempered by a desire to see an adjustment in the balance between curiosity-driven research and priority-driven research. The health service leaders whom we interviewed identified several practical ways of achieving progress. They included providing more (and more appropriate) support for research that was better aligned with the priorities of health services; overcoming organisational barriers that inhibit interactions between researchers and the users of research; and building the capacity and capability of health staff to do and use research. These measures should help to embed health services research more systematically in health service organisations and make it an essential and integrated part of health care delivery.

## Data Availability

The interview recordings and notes are safely stored on a secure Sax Institute server only accessible via a password protected compute to protect the participants’ identities.

## References

[1] Hanney SR, González-Block MA (2015). Health research improves healthcare: now we have the evidence and the chance to help the WHO spread such benefits globally. Health Res Policy Syst.

[2] Global Commission on Evidence to Address Societal Challenges. The evidence commission report: a wake-up call and path forward for decision-makers, evidence intermediaries, and impact-oriented evidence producer. Hamilton, Canada; 2022. https://www.mcmasterforum.org/docs/default-source/evidence-commission/evidence-commission-report.pdf?sfvrsn=2fb92517_11. Accessed 28 Nov 2023.

[3] Morris ZS, Wooding S, Grant J (2011). The answer is 17 years, what is the question: understanding time lags in translational research. J R Soc Med.

[4] Braithwaite J, Glasziou P, Westbrook J (2020). The three numbers you need to know about healthcare: the 60-30-10 Challenge. BMC Med.

[5] Reed JE, Howe C, Doyle C, Bell D (2018). Simple rules for evidence translation in complex systems: a qualitative study. BMC Med.

[6] Greenhalgh T, Papoutsi C (2018). Studying complexity in health services research: desperately seeking an overdue paradigm shift. BMC Med.

[7] Braithwaite J, Churruca K, Long JC, Ellis LA, Herkes J (2018). When complexity science meets implementation science: a theoretical and empirical analysis of systems change. BMC Med.

[8] Robinson T, Bailey C, Morris H, Burns P, Melder A, Croft C (2020). Bridging the research–practice gap in healthcare: a rapid review of research translation centres in England and Australia. Health Res Policy Syst.

[9] Oborn E, Barrett M, Racko G (2013). Knowledge translation in healthcare: incorporating theories of learning and knowledge from the management literature. J Health Organ Manag.

[10] Chalmers I, Glasziou P (2009). Avoidable waste in the production and reporting of research evidence. Lancet.

[11] Lomas J, Fulop N, Gagnon D, Allen P (2003). On being a good listener: setting priorities for applied health services research. Milbank Q.

[12] Figueroa CA, Harrison R, Chauhan A, Meyer L (2019). Priorities and challenges for health leadership and workforce management globally: a rapid review. BMC Health Serv Res.

[13] Toh CH, Haynes R (2022). The health and care act 2022: challenges and priorities for embedding research in the NHS. Lancet.

[14] Anderson S, Allen P, Peckham S, Goodwin N (2008). Asking the right questions: scoping studies in the commissioning of research on the organisation and delivery of health services. Health Res Policy Syst.

[15] Greenhalgh T, Jackson C, Shaw S, Janamian T (2016). Achieving research impact through co-creation in community-based health services: literature review and case study. Milbank Q.

[16] Rycroft-Malone J, Burton CR, Bucknall T, Graham ID, Hutchinson AM, Stacey D (2016). Collaboration and co-production of knowledge in healthcare: opportunities and challenges. Int J Health Policy Manag.

[17] McKeon S. Strategic review of health and medical research in Australia—better health through research. Canberra; 2013. https://cheba.unsw.edu.au/sites/cheba2/files/blog/pdf/Strategic_Review_of_Health_and_Medical_Research_Feb_2013-Summary_Report.pdf. Accessed 28 Nov 2023.

[18] Australian Academy of Health and Medical Sciences. Research and innovation as core functions in transforming the health system: a vision for the future of health in Australia. 2022. https://cheba.unsw.edu.au/sites/cheba2/files/blog/pdf/Strategic_Review_of_Health_and_Medical_Research_Feb_2013-Summary_Report.pdf. Accessed 28 Nov 2023.

[19] Agency for Healthcare Quality and Research (AHQR). 2023. https://www.ahrq.gov/. Accessed 28 Nov 2023.

[20] National Institute for Health and Care Research. 2023. https://www.nihr.ac.uk/. Accessed 28 Nov 2023.

[21] Australian Government. Medical Research Future Fund 2023. https://www.health.gov.au/our-work/medical-research-future-fund. Accessed 28 Nov 2023.

[22] Hoekstra F, Mrklas K, Khan M, McKay R, Vis-Dunbar M, Sibley K (2020). A review of reviews on principles, strategies, outcomes and impacts of research partnerships approaches: a first step in synthesising the research partnership literature. Health Res Policy Syst.

[23] Gagliardi AR, Berta W, Kothari A, Boyko J, Urquhart R (2016). Integrated knowledge translation (IKT) in health care: a scoping review. Implement Sci.

[24] Abu-Odah H, Said NB, Nair SC, Allsop MJ, Currow DC, Salah MS (2022). Identifying barriers and facilitators of translating research evidence into clinical practice: a systematic review of reviews. Health Soc Care Community.

[25] Moore G, Redman S, Rudge S, Haynes A (2018). Do policy-makers find commissioned rapid reviews useful?. Health Res Policy Syst.

[26] Black AT, Steinberg M, Chisholm AE, Coldwell K, Hoens AM, Koh JC (2021). Building capacity for implementation-the KT Challenge. Implement Sci Commun.

[27] Tait H, Williamson A (2019). A literature review of knowledge translation and partnership research training programs for health researchers. Health Res Policy Syst.

[28] Tong A, Synnot A, Crowe S, Hill S, Matus A, Scholes-Robertson N (2019). Reporting guideline for priority setting of health research (REPRISE). BMC Med Res Methodol.

[29] Sydney Health Partners. 2023. https://sydneyhealthpartners.org.au/. Accessed 28 Nov 2023.

[30] Gale NK, Heath G, Cameron E, Rashid S, Redwood S (2013). Using the framework method for the analysis of qualitative data in multi-disciplinary health research. BMC Med Res Methodol.

[31] Tong A, Sainsbury P, Craig J (2007). Consolidated criteria for reporting qualitative research (COREQ): a 32-item checklist for interviews and focus groups. Int J Qual Health Care.

[32] Atkins D, Kilbourne AM, Shulkin D (2017). Moving from discovery to system-wide change: the role of research in a learning health care system: experience from three decades of health systems research in the Veterans Health Administration. Annu Rev Public Health.

[33] King's Health Partners. 2023. https://www.kingshealthpartners.org/. Accessed 28 Nov 2023.

[34] Australian Government, Department of Health and Aged Care. Improving alignment and coordination between the Medical Research Future Fund and Medical Research Endowment Account. 2023. https://consultations.health.gov.au/health-economics-and-research-division/improving-alignment-and-coordination-mrff-mrea/. Accessed 28 Nov 2023.

[35] Ritchie J, Spencer L, Bryman A, Burgess R (1994). Qualitative data analysis for applied policy research. Analyzing qualitative data.

